# Non-Covalent Forces in Naphthazarin—Cooperativity or Competition in the Light of Theoretical Approaches

**DOI:** 10.3390/ijms22158033

**Published:** 2021-07-27

**Authors:** Aneta Jezierska, Kacper Błaziak, Sebastian Klahm, Arne Lüchow, Jarosław J. Panek

**Affiliations:** 1 Faculty of Chemistry, University of Wrocław, ul. F. Joliot-Curie 14, 50-383 Wrocław, Poland; jaroslaw.panek@chem.uni.wroc.pl; 2 Faculty of Chemistry, University of Warsaw, ul. Pasteura 1, 01-224 Warsaw, Poland; 3 Biological and Chemical Research Center, University of Warsaw, Żwirki i Wigury 101, 01-224 Warsaw, Poland; 4 Institute of Physical Chemistry, RWTH Aachen University, 52056 Aachen, Germany; Sebastian.Klahm@rwth-aachen.de (S.K.); luechow@pc.rwth-aachen.de (A.L.)

**Keywords:** Naphthazarin C, 5,8-dihydroxynaphthalene-1,4-dione, hydrogen bond, gas phase, crystalline phase, DFT, MP2, DQMC, CC, HOMA index, Fukui function, AIM, SAPT, CPMD

## Abstract

Non-covalent interactions responsible for molecular features and self-assembly in Naphthazarin C polymorph were investigated on the basis of diverse theoretical approaches: Density Functional Theory (DFT), Diffusion Quantum Monte Carlo (DQMC), Symmetry-Adapted Perturbation Theory (SAPT) and Car-Parrinello Molecular Dynamics (CPMD). The proton reaction paths in the intramolecular hydrogen bridges were studied. Two potential energy minima were found indicating that the proton transfer phenomena occur in the electronic ground state. Diffusion Quantum Monte Carlo (DQMC) and other levels of theory including Coupled Cluster (CC) employment enabled an accurate inspection of Potential Energy Surface (PES) and revealed the energy barrier for the proton transfer. The structure and reactivity evolution associated with the proton transfer were investigated using Harmonic Oscillator Model of Aromaticity - HOMA index, Fukui functions and Atoms In Molecules (AIM) theory. The energy partitioning in the studied dimers was carried out based on Symmetry-Adapted Perturbation Theory (SAPT) indicating that dispersive forces are dominant in the structure stabilization. The CPMD simulations were performed at 60 K and 300 K in vacuo and in the crystalline phase. The temperature influence on the bridged protons dynamics was studied and showed that the proton transfer phenomena were not observed at 60 K, but the frequent events were noticed at 300 K in both studied phases. The spectroscopic signatures derived from the CPMD were computed using Fourier transformation of autocorrelation function of atomic velocity for the whole molecule and bridged protons. The computed gas-phase IR spectra showed two regions with OH absorption that covers frequencies from 2500 cm${}^{-1}$ to 2800 cm${}^{-1}$ at 60 K and from 2350 cm${}^{-1}$ to 3250 cm${}^{-1}$ at 300 K for both bridged protons. In comparison, the solid state computed IR spectra revealed the environmental influence on the vibrational features. For each of them absorption regions were found between 2700–3100 cm${}^{-1}$ and 2400–2850 cm${}^{-1}$ at 60 K and 2300–3300 cm${}^{-1}$ and 2300–3200 cm${}^{-1}$ at 300 K respectively. Therefore, the CPMD study results indicated that there is a cooperation of intramolecular hydrogen bonds in Naphthazarin molecule.

## 1. Introduction

The characteristics of non-covalent interactions in Naphthazarin (5,8-dihydroxynaphthalene-1,4-dione), in particular its C polymorph, are an object of the current study. As it is well-known, non-covalent interactions are ubiquitous in Nature [1,2]. They determine various processes in chemistry, physics and in biodisciplines. Comparing covalent bonds with intra- and intermolecular non-covalent interactions, one can see that the latter are weaker, exhibit lower energy and directionality. However, as it is well-known the interactions collectively play a dominant role in many processes at molecular level, e.g., the nucleic acids constitution [3,4]. In fact, the flexibility of non-covalent interactions makes them well-suited to serve as principal constituents of dynamical systems (i.e., liquid phase and living organisms), where rigidity would prevent the system from actual functioning. The nature and examples of non-covalent forces were an object of many review articles e.g., Refs. [5,6,7,8,9]. The external forces could be neglected only for isolated molecule, but when the molecule is surrounded by other molecules, such as in solution or in the bulk, the neighbourhood of other molecules affects the covalent bonding and the electronic structure of the molecule. The introduced changes in the molecule depend on the strength and extent of non-covalent interactions. In some cases, they could be comparable with covalent interactions, e.g., changes occurring in ionic and H-bonded systems [6,10]. The non-covalent interactions differ from covalent bonds, because they do not involve the electron sharing as a primary binding factor. Moreover, they occur between molecules rather than between different atoms of the same molecule. They could be classified into different categories: electrostatic, π effects, van der Waals forces and hydrophobic effects [11]. Among electrostatic interactions one can find ionic interactions, hydrogen and halogen bonding [12,13]. The van der Waals interactions cover London dispersion forces, dipole-dipole and dipole-induced dipole. The π effects are associated with the interactions of molecules containing π electrons. They comprise π-π interaction, polar-π and cation-π and anion-π interactions [14]. The last is the hydrophobic effect, which is desired for non-polar molecules to aggregate in aqueous solutions and separate from water [15]. Non-covalent interactions are non-substitutable as driving forces to stabilize the three-dimensional structure of macromolecules, such as proteins and nucleic acids. One has to remember that they are involved in many processes where molecular complexes are formed, e.g., large molecules bind specifically to one another or they interact with small ligands. Summarizing, non-covalent forces heavily influence the synthesis of many organics molecules, they cannot be neglected in drug design, crystallinity and design of materials—particularly for self-assembly [2,16] and formation of cocrystals [17].

Naphthazarin, 5,8-dihydroxynaphthalene-1,4-dione, contains intra- and intermolecular hydrogen bonds [18]. The hydrogen bonding is classified as a resonance-assisted hydrogen bond (RAHB) [19]. The presence of such type of interactions was found to be significant in various processes in chemistry, biology and material science, as it has been shown by selected examples [12,20,21,22]. The crystal structure of Naphthazarin C was determind using neutronography (at 60 K and 300 K) and X-ray diffraction (at 300 K) [18]. It was found that depending on the temperature the space groups are Pc at 60 K, but P21/c at 300 K. It is important to stress that neutron diffraction measurements showed that the Pc and P21/c structures are related by an order-disorder transition at 110 ± 1 K. The structure with C${}_{2v}$ symmetry is preferred at 60 K therefore the appropriate molecular formula should be 5,8-dihydroxy-1,4-naphthalene-1,4-dione. However, it is important to notice that neutron diffraction as well as X-ray diffraction measurements at 300 K on Naphthazarin C indicated a disordered molecular model with one-half of an hydrogen atom attached to each oxygen. The polymorphs A and B also support the model. Further comparisons of polymorphs A, B and C showed that the O-H...O intermolecular hydrogen bond was detected in the latter structure. The crystallographic analysis combined with available solid state NMR data provided information that at room temperature there is a rapid intramolecular proton exchange in the hydrogen bridge in all three Naphthazarin polymorphs [23,24,25,26]. The IR and Raman spectra were recorded for Naphthazarin confirming the presence of the intramolecular hydrogen bonds. The experimentally obtained data was supported by quantum-chemical simulations. It was found in the vibrational analysis that a strong coupling exists between the modes of chelated ring. The centers of υOH/OD and γOH/OD stretching modes were identified in Naphthazarin at 3060/2200 cm${}^{-1}$ and 793/560 cm${}^{-1}$ respectively [27]. The absorption spectra of Naphthazarin were investigated experimentally and theoretically as well, because the molecule belongs to the class of quinoid dyes. Jacquemin et al. demonstrated that the specific shape of the experimental band cannot be due to multi-absorptions nor aggregation of molecules nor due to the presence of tautomers [28]. The previous studies showed that Naphthazarin is a molecule interesting and worth further consideration. Therefore, we employed diverse theoretical approaches to shed more light onto non-covalent forces determining structure and features of the system. Let us focus on particular non-covalent forces-hydrogen bonds. There have been many efforts to understand and describe hydrogen bonding nature and its role in many processes occurring at molecular level. Nevertheless, there are still some open questions related to the proton transfer phenomenon, the strength of the interaction or to the environmental effects influence on the hydrogen bonding features. The steric and inductive effects play an important role in the modulation of the hydrogen bridge [29,30] determining the bridged proton position. The presence of the intramolecular hydrogen bond is associated with the quasi-ring formation and further with the internal reorganization of the geometric parameters and electronic structure. Concerning the structural chemistry, the formation of the hydrogen bond is able to stabilize the system, but on the other hand, it affects parts of molecules changing not only the geometric parameters and the distribution of the electron density, but also the aromaticity (the changes of the π electrons delocalization) of the aromatic rings [12,31]. Therefore, the consequences of the hydrogen bond presence could be classified as short and long range interactions. In Naphthazarin molecule our attention was focused on non-covalent intra- and intermolecular forces. Therefore, our computational study involved the investigations of isolated molecule, examples of dimers found in the crystal structure and finally - the molecular crystal of the C polymorph [18]. Naphthazarin, it is worth to underline, has been an object of many experimental and theoretical studies due to its molecular structure and diverse features derived from the arrangement of two short intramolecular hydrogen bridges (HB) connected by two fused rings, e.g., Refs. [18,24,27,28,32,33,34,35,36,37,38,39,40]. These bridges are classified as strong and enhanced by resonance effects as well as spatial proximity of the donor and acceptor moieties enforced by the rigid naphthalene skeleton of aromatic rings [18,19,34]. Since the two HBs could be potentially coupled by the aromatic skeleton, the routes of modulation of their properties including substitution in the aromatic ring as well as changes of the system size (number of rings) have been studied in the light of various potential practical application of Naphthazarin derivatives, e.g., [41,42,43,44,45,46]. In the current study using various theoretical approaches we investigated: (i) for the Naphthazarin monomeric forms, the proton potential functions with special attention paid to the height of the energy barrier necessary for the proton transfer (PT) phenomenon—the Quantum Monte Carlo approach [47] was included in this part to provide accurate barrier height; (ii) the thermodynamic reaction path for the proton motion; (iii) aromaticity evolution as a function of the $H^{BP1}$...O1 distance changes using Harmonic Oscillator Model of Aromaticity (HOMA) index; (iv) changes in the reactivity of molecular sites on the basis of Fukui functions [48]; (v) the intermolecular forces responsible for the dimers stabilization using Symmetry-Adapted Perturbation Theory (SAPT) [49]; and finally we employed Car-Parrinello Molecular Dynamics (CPMD) approach [50] to study in detail: (vi) the bridged proton mobility in the gas phase (isolated molecule model) and in the crystalline phase to detect cooperativity or competition of various forces influencing the dynamics of Naphthazarin molecule, in particular the question of time sequence of the double proton transfer; (vii) the insight into vibrational features using the Fourier transformation of the time autocorrelation function of the atomic velocity [51]. Therefore, we can define as one of the aims of the study the development of interaction models covering various factors significant in the structure arrangement and molecular self-assembly. In addition, we discuss the cooperativity/competition of non-covalent forces present in Naphthazarin using diverse theoretical approaches to make our study detailed and comprehensive. Up to our knowledge this is the first study discussing non-covalent forces in monomeric and dimeric forms of Naphthazarin as well as in the crystalline phase. In addition, for the first time the Diffusion Quantum Monte Carlo and CPMD approaches were employed to revealed molecular features in Naphthazarin C.

## 2. Results and Discussion

### 2.1. Naphthazarin Monomer Non-Covalent Intramolecular Interactions Study

**Figure 1:**
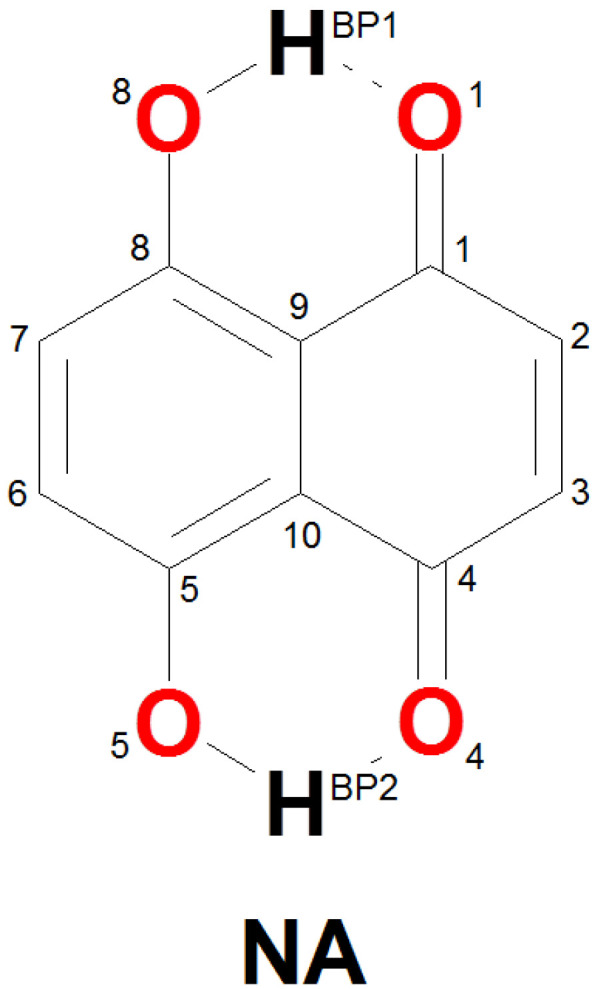
Molecular form of Naphthazarin with atoms numbering scheme applied in the study. The dotted lines indicate the presence of intramolecular hydrogen bonds.

**Figure 2:**
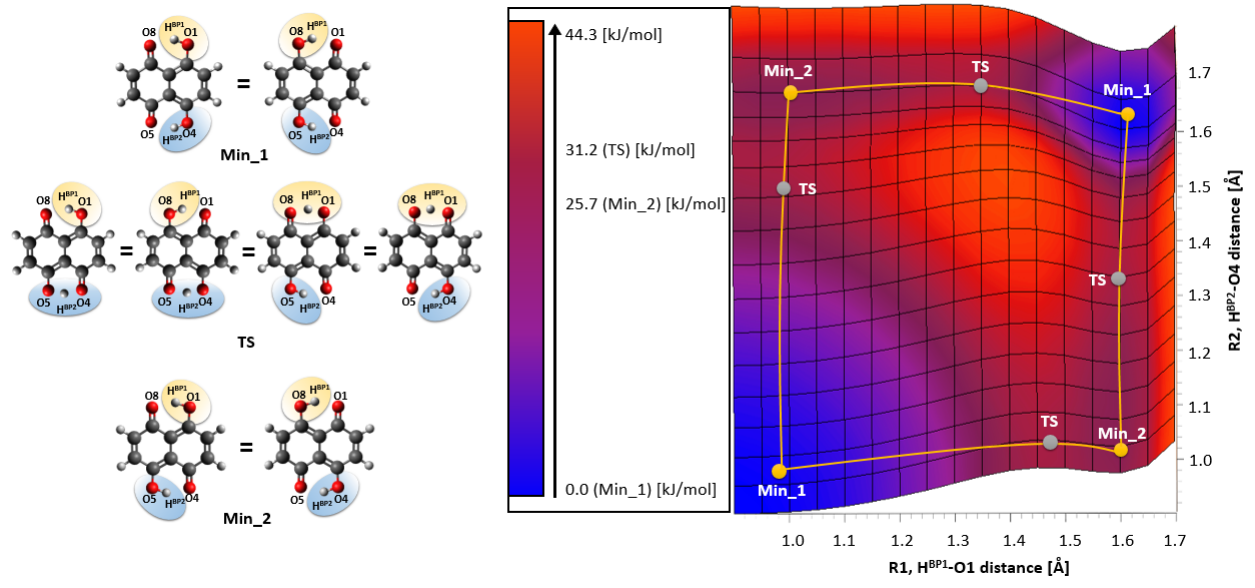
Two-dimensional potential energy surface (2D PES) for both proton motions between the donor and acceptor atoms (R1, H${}^{BP1}$-O1 and R2, H${}^{BP2}$-O4 bond distances) computed at CAM-B3LYP/6-311+G(2d,2p) level of theory (left). The corresponding isomeric structures indicated on the surface (right). Min_1—C${}_{2v}$ energy minimum with the plane symmetry; Min_2—C${}_{2h}$ energy minimum; TS—transition state.

**Figure 3:**
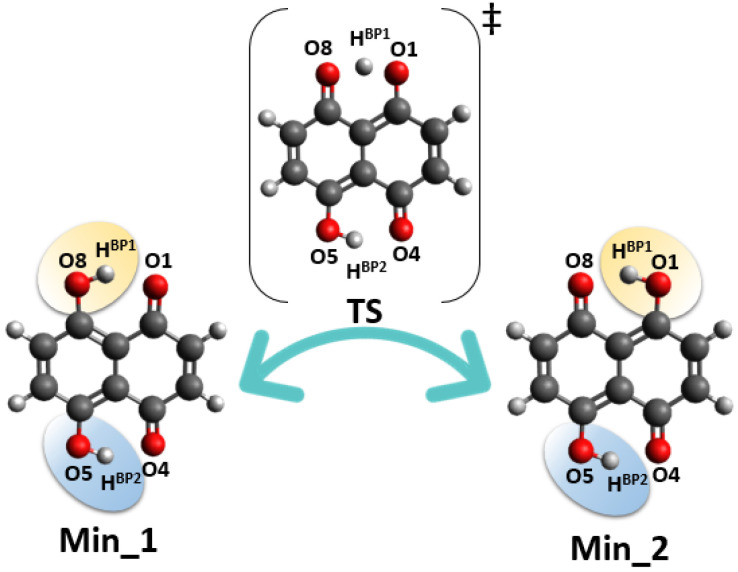
Proton transfer reaction model of Naphtazarin. Min_1—C${}_{2v}$ energy minimum with the plane symmetry; Min_2—C${}_{2h}$ energy minimum; TS—transition state.

The molecular form of Naphthazarin and its tautomers are presented in Figure 1 and Figure S1 respectively. The intramolecular hydrogen bond proton reaction paths were studied on the basis of DFT using CAM-B3LYP/6-311+G(2d,2p) level of theory. The obtained results are presented in Figure 2. As it is shown two energy minima were detected on the potential energy surface (PES). They are denoted as Min_1 (corresponds with C${}_{2v}$ symmetry structure) and Min_2 (corresponds with C${}_{2h}$ symmetry structure) respectively. The C${}_{2v}$ symmetry structure is thermodynamically preferable, which could be related to cooperativity of the intramolecular non-covalent forces. The OH groups position in Naphthazarin is according to our expectations and in agreement with previous findings reported in the literature [34,40,52]. Next, we have studied the thermodynamics of the reaction path of single (asynchronous) proton transfer based on DFT and MP2 methods. The proton transfer (PT) reaction model used to estimate changes in the total energy (ΔE), enthalpy (ΔH) and free Gibbs energy (ΔG) is presented in Figure 3 whereas the relative values of thermodynamical properties are presented in Table 1. The values were obtained in relation to Min_1. In order to estimate the Min_1, transition state (TS) and the second Min_2 energy, six hybrid functionals were employed with MP2 method as a reference. The TPSSh functional provided the lowest energy barrier and secondary minimum position for TS and the Min_2 respectively. The highest energy values were obtained for the M08-HX functional. The energy difference between the TPSSh functional and M08-HX is 21 kJ/mol for TS and 15.5 kJ/mol concerning the Min_2. The MP2 method estimated the energy as 28.2 kJ/mol for TS and 21.3 kJ/mol for the second minimum. As we can see only the performance of M08-HX functional could not be satisfactory, because the obtained energy values are much higher comparing with MP2 method and other applied functionals. The values of enthalpy and Gibbs free energy were reproduced with the hierarchy observed for the total energy. No other significant changes were noticed in the thermodynamic characteristics of the proton reaction path in Naphthazarin, signifying that (at least for the isolated molecule) rotational and vibrational enthalpic-entropic corrections are rather similar over the studied region of PES. Therefore, we can conclude that the bridged protons could move between the proton-donor and acceptor atoms in the hydrogen bridge, because the computed thermodynamic characteristics showed that such a process is possible with relatively small barriers. It is worth to underline that our findings are in agreement with previously reported data concerning the proton mobility in the Naphthazarin molecule [40,53,54].

Following the discussion of the thermodynamics of the bridged protons mobility, the energy barrier for the double (synchronous) proton transfer was computed at various levels of theory including Diffusion Quantum Monte Carlo (DQMC) method [47]. The synchronous proton transfer was found not to be the preferred proton motion mode, however it is an interesting process on its own. It requires more de-aromatization than the asynchronous PT, and it puts more stress on the performance of the electron correlation methods, thus being an ideal benchmark of computational levels. We have focused on mainly two structures: in the energy minimum (C${}_{2v}$ symmetry structure) and the D${}_{2h}$ second-order saddle point (SSP, the protons localized in the middle of the hydrogen bridges), for clarity see Figure S2. The obtained results are presented in Table 2. As it is shown, the employment of various levels of theory provided a broad spectrum of the performance of theoretical approaches. The lowest energy barrier was obtained for B3LYP/TZPAE//MP2/aug-cc-pVTZ, MP2/CBS//MP2/cc-pVTZ and MP2/aug-cc-pVTZ//MP2/cc-pVTZ levels of theory. An application of Quantum Monte Carlo approach provided an accurate Potential Energy Surface (PES) characteristics, which resulted with energy barrier of 60.26 ± 1.14 kJ/mol. The obtained value is much higher comparing to the data reported for DFT and MP2 methods. As it is shown an application of Coupled Cluster (CC) methods as a result gave an energy barrier range between 50.97 kJ/mol–69.07 kJ/mol. The last part of the Table 2 reports on results obtained for trans position of the OH groups in Naphthazarin. They are in good agreement with our DFT and MP2 findings reported in Table 1. The most important observation from this part of study is that, when barrier height data from Table 1 and Table 2 are compared, the synchronous double PT event is indeed less probable, but not excluded. This could be true especially for the dynamical scenario [55], where the thermal kinetic energy of the molecule could allow the SSP transversal of the PES plateau seen in Figure 2. The CPMD study will shed light on the possibility of such an event.

**Figure 4:**
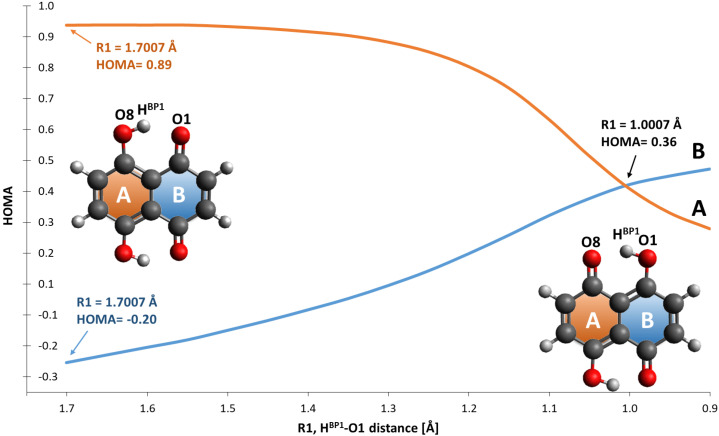
HOMA index evolution as a function of the intramolecular hydrogen bond distance changes.

**Figure 5:**
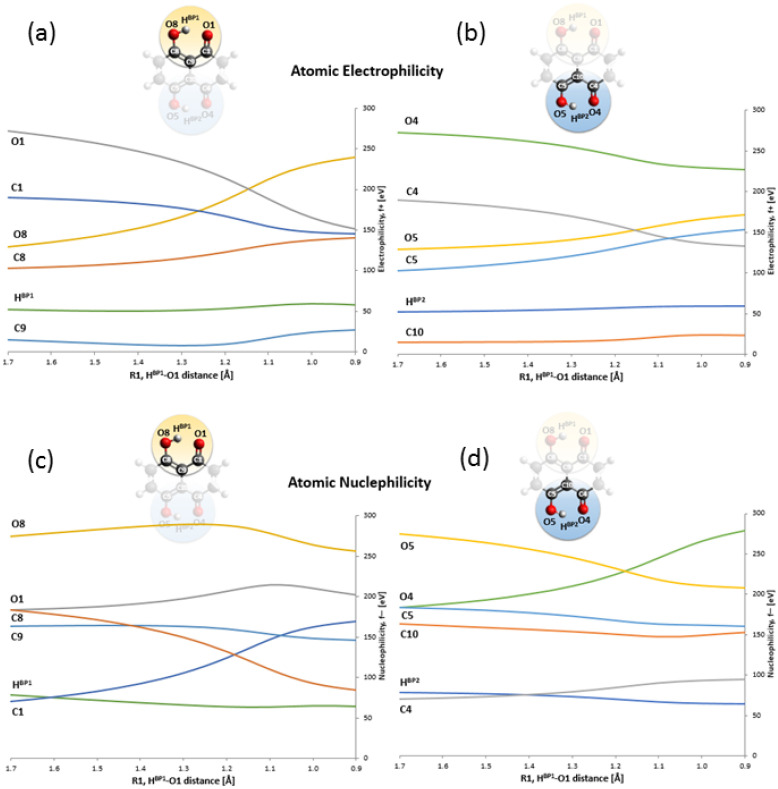
Atomic electrophilicity and nucleophilicity evolution as a function of the intramolecular hydrogen bond distance changes. Atomic electrophilicity for the (**a**) O8...O1 and (**b**) O5...O4 bridge, and atomic nucleophilicity for the (**c**) O8...O1 and (**d**) O5...O4 bridge are presented.

The static models results confirmed that the energy barriers make possible the PT event in Naphthazarin molecule. The bridged protons dynamics is strongly related with the internal geometric parameters as well as electronic structure reorganization [56]. The presence of intramolecular hydrogen bonds resulted in formation of two quasi-rings, which are responsible for additional intramolecular interactions, e.g., stronger stabilization of the hydrogen-bonded form of Naphthazarin. The consequences of the intramolecular structure reorganization upon the bridged proton position we have analyzed using descriptors such as HOMA aromaticity index [57] and Fukui functions [48]. The HOMA aromaticity index enables quantitative estimation of the aromaticity changes dependent on the proton position in the hydrogen bridge. Naphthazarin molecule consists of two fused rings—benzene and 1,4-benzoquinone. The aromaticity changes introduced by the bridged proton mobility are presented in Figure 4. When the bridged proton is moving to the proton-acceptor atom (O1) the aromaticity is decreasing in the ring A from 0.89 (HOMA index) to 0.23 while the proton reaches the acceptor atom. An opposite situation was noticed for the ring B. When the bridged proton is reaching the proton-acceptor the aromaticity in the ring B is increasing significantly from −0.2 to 0.42. It is a quantitative evidence of the intramolecular features reorganization associated with the presence of cooperativity of the non-covalent intramolecular interactions. We have applied the Fukui functions [58,59] to follow the electrophilicity and nucleophilicity changes related to the bridged proton position on the one site of the molecule from global minimum (Min_1) to the intermediate structure (Min_2). The atoms involved in the quasi-rings formation were taken into consideration as showed in Figure 5. The CAM-B3LYP/6-311+G(2d,2p) level of theory was applied to study the electronic structure changes as a function of the $H^{BP1}$...O1 distance changes. As it is shown the significant changes of electrophilicity are visible for four atoms (O1, C1, C8 and O8) in the quasi-ring consisting of atoms (O1, C1, C9, C8, O8 and H${}^{BP1}$). The electrophilicity decreases in case of C1 from 272.2 to 151.6 eV and O1 atoms from 189.9 to 146.6 eV, but it increases concerning C8 (from 103.1 to 239.6 eV) and O8 (from 129.4 to 239.6 eV) atoms while the bridged proton is moving to the acceptor side. The Naphthazarin molecule is symmetric and one could expect that in both quasi-rings the changes of electrophilicity will be equal, however as a result of the one-site proton scan the second intermediate (Min_2) structure is formed posing C${}_{2}h$ symmetry. On the other hand, the conceptual parameters: nucleophilicity and electrophilicity are not their quantitative mirror reflection and usually do not compensate each other while the reaction is occurring [60]. As we can see (in Panel B of Figure 5) a similar tendency is noticed, while quantitatively the situation is different, due to both facts that the molecule has changed into asymmetric second intermediate and that the electron density is only partially reorganized. The electrophilicity decreases for O4 and C4 atoms (from 272.2 to 227.1 eV and from 189.9 to 133.3 eV, respectively), but it increases for O5 and C5 atoms (from 130.1 to 172.0 eV and from 103.1 to 153.6 eV, respectively). In both quasi-rings the C9 and C10 atoms do not show large changes in the electrophilicity (they exhibit rather slight increase). Most probably, because they are shielded by other atoms in the quasi-rings. The electrophilicity of the protons does not depend on their position in the hydrogen bridge. The changes of nucleophilicity are presented in lower panels of Figure 5. As we could expect the observed tendency has been reversed: for O8, C8 and O5, C5 the nucleophilicity increases while for O1, C1 and C4, O4 decreases. In C9, C10 atoms rather a slight decrease of the nuclephilicity was observed. Concerning the bridged proton $H^{BP1}$ and $H^{BP2}$ a slight decrease of the nucleophilicity was noticed. Concluding, the Fukui functions analysis described the electron density in a frontier orbitals changes, as a result of a small change in the total number of electrons upon the bridged protons movement. This gave an insight into electronic structure changes as a result of an internal reorganization of the molecule while the bridged protons are moving between the donor and acceptor atoms on the one side of the molecule, as an intramolecular transition between intermediates Min_1 and Min_2.

**Figure 6:**
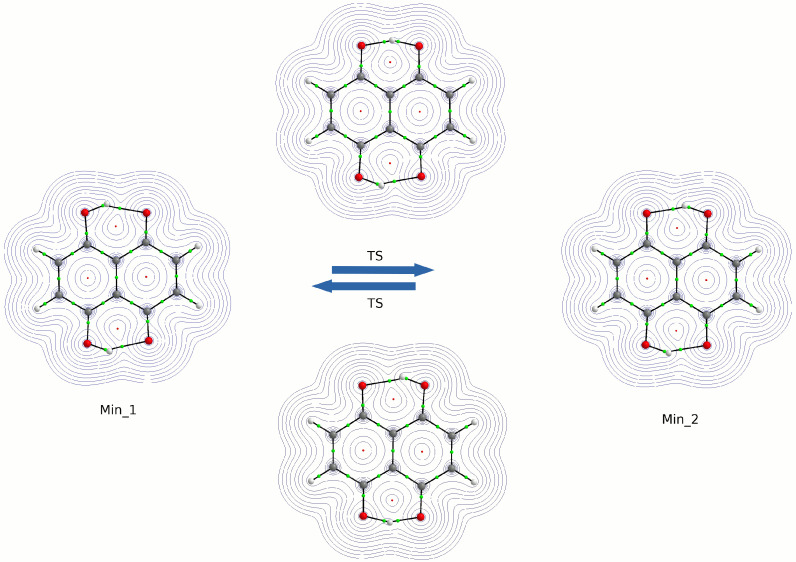
AIM topology maps of the proton transfer reaction model of Naphthazarin on the basis of CAM-B3LYP/6-311+G(2d,2p) level of theory. The black solid and dashed lines indicate the presence of intramolecular interactions. Green and red dots mark the presence of BCPs and RCPs detected by the analysis.

The Atoms In Molecules (AIM) topology maps of electron density of Naphthazarin are presented in Figure 6. On the basis of the theory the thermodynamic reaction path topological details were reproduced. The presence of the covalent and hydrogen bonds was detected by the Bond Critical Points (BCPs). The fused rings and formed—by the presence of intramolecular hydrogen bonds—quasi-rings were indicated by Ring Critical Points (RCPs). An interesting feature of the electron density topology maps is whether the located critical points are considered “stable” or “metastable”. In case of weak intramolecular interactions, the relevant BCP and RCP can be located in close proximity, so that even a small structural modification would lead to the coalescence of these two topological features [61]. This is not the case for Naphthazarin at various stages of the PT event, indicating that the hydrogen bridges remain strong, important structural features of the molecule even at the PT transition state structure. The obtained results are in agreement with experimental (neutron diffraction and X-ray [18]) structural studies confirming the chemical composition of the molecule. Further, the properties of the electron density at the hydrogen bridge proton-acceptor BCP have been used as indicators of the hydrogen bond strength. This is important especially when intramolecular bonding is analyzed, where various factors make assignment of the hydrogen bond energy difficult or ambiguous—this fact has been reviewed recently [62,63]. We have followed the formulas of Espinosa [64] and Vener [65], which state, respectively, that E${}_{HB}$ = −V(r)/2 and E${}_{HB}$ = 0.429 G(r) (the potential V(r) and Lagrangian kinetic G(r) energy densities are taken from the bridge BCP). These formulas were recently used for non-conventional C-H...halogen and B-H...π bonds [66,67,68]. In case of Naphthazarin, the corresponding bridge BCP parameters are: V(r) = −0.0437 Hartree and G(r) = 0.03885 Hartree, which yields the HB strength estimates of 13.7 and 10.5 kcal/mol. These values cannot be directly compared to the barrier heights from Table 1 and Table 2, because the proton transfer process involves large structural rearrangement and loss of aromaticity in the bicyclic moiety, heightening the energetic cost. From this point of view, the obtained estimates of the HB strength seem very reasonable.

### 2.2. Intermolecular Interactions in Dimers of Naphthazarin in the Light of Symmetry-Adapted Perturbation Theory (SAPT)

**Figure 7:**
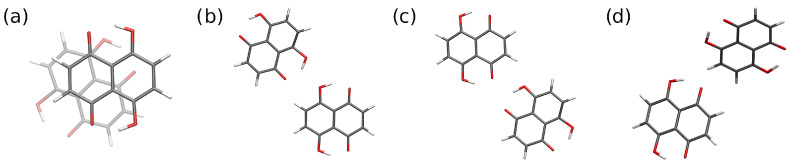
Dimers of Naphthazarin selected for the interaction energy analysis. Structures taken from the 60 K neutron diffraction crystal structure [18]. Dimers represent the following interactions: (**a**) stacking, (**b**) and (**c**) hydrogen bonding, (**d**) multipole interaction and possible weak C-H...H bonds.

Details of crystal packing are governed by subtle interplay between diverse types of intermolecular forces. This is also true for Naphthazarin. Its aromatic skeleton introduces dispersion forces, while both hydroxyl and carbonyl functions are polar and capable of hydrogen bond formation. Symmetry-Adapted Perturbation Theory [49] was chosen as the framework to discuss the structural role of different kinds of interactions. We have selected four types of dimers (see Figure 7) present in the experimental structure of Naphthazarin. The resulting interaction energy partitioning is presented in Table 3 for the experimental structures as well as for the DFT optimization results.

The SAPT results indicate the primary significance of dispersion interactions in the formation of the strongest structural motif, the stacked dimer. On the other hand, this is also the interaction most susceptible to structural modifications. While the SAPT2 interaction energy is −15.88 kcal/mol for the experimental structure, it is only −10.69 kcal/mol for the DFT-optimized dimer. In none of the other studied dimers such difference would exceed 1 kcal/mol, indicating structural rigidity of these dimers. The energy term that changes most is, however, exchange contribution. Strong repulsion (19.73 kcal/mol) at the experimental geometry falls down to 4.72 kcal/mol after the DFT optimization. This means that the solid state structure is relatively compressed and the molecules in the stack are much closer than in the gas-phase dimer. On the other hand, this compression—and relatively large exchange energy—is also the basis of formation of the solid state properties, since large exchange contribution indicates also significant orbital overlap. This is also visible in the $\delta E_{HF}^{\left(2\right)}$ term, which is largest in the experimental stacked structures—indicating deviations from the “lack of orbital overlap” condition, under which the perturbational techniques similar to SAPT perform best. The hydrogen-bonded dimers are much more rigid structurally and their interaction energies are similar between the experimental and DFT-optimized structures. It is important to note that the electrostatic-driven interaction in the dimer type (d), possibly combined with weak C-H...H bonds, was able to be retained in the gas phase calculations. Thus, Naphthazarin, an aromatic system with symmetric substitution providing less opportunity for electrostatics-driven dimer formation, arranges its crystal structure so that the stacking (dispersion) forces are of primary importance, and the Coulomb forces are secondary with respect to the formation of structural motifs.

### 2.3. Intramolecular Hydrogen Bonds Geometric and Vibrational Properties Investigated Based on Car-Parrinello Molecular Dynamics (CPMD) in the Gas and Crystalline Phases

**Figure 8:**
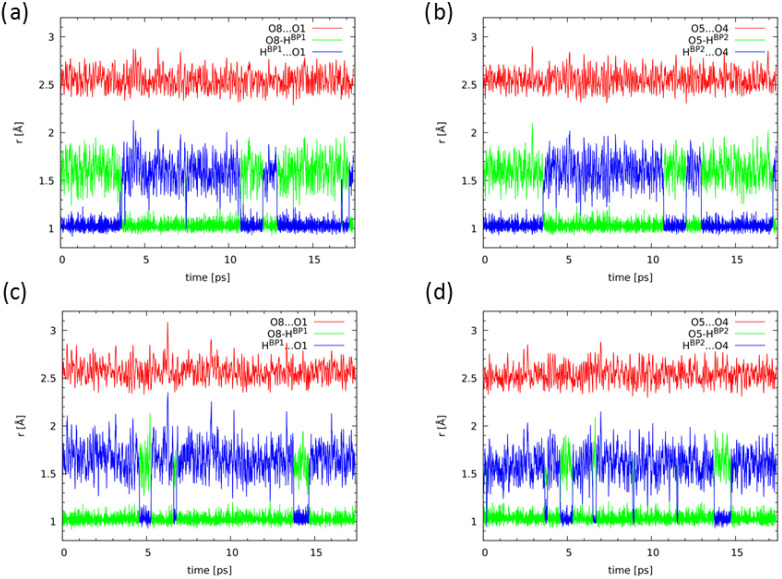
Time evolution of the metric parameters of the hydrogen bridges. Results of the CPMD simulation at 300 K: (**a**) gas phase, bridge 1; (**b**) gas phase, bridge 2; (**c**) solid state, bridge 1; (**d**) solid state, bridge 2.

**Figure 9:**
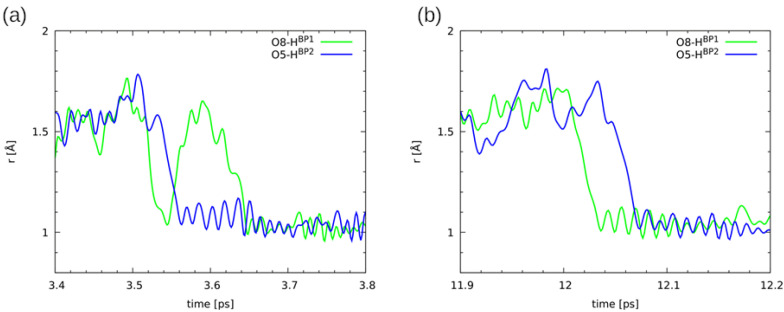
Details of two events of double proton transfer from the donor to the acceptor side—close-ups of the gas-phase CPMD trajectory at the room temperature at (**a**) 3.5 ps and (**b**) 12 ps of the simulation time. Both bridge protons are monitored so that the degree of correlation between their motions can be assessed.

Starting from optimized molecular structure of Naphthazarin C, Car-Parrinello Molecular Dynamics (CPMD) simulations were performed at 60 K and 300 K in vacuo and in the crystalline phase. The time-evolution of hydrogen bridges is presented in Figure S5 and Figure 8 respectively. Let us start the discussion of the CPMD results obtained for the gas phase simulations at 60 K. As it is shown in Figure S5 (panels a and b) the OH distances have not changed significantly during ca. 25 ps simulations time. Both bridged protons are localized on the donor side (atoms O8 and O5 respectively). A completely different picture of the bridged protons behaviour was obtained as a result of the CPMD simulations at 300 K. It is evident that the O8-H${}^{BP1}$ and O5-H${}^{BP2}$ covalent bonds length significantly were changing during the simulation time. Figure 8, panels a and b, illustrates the bridged protons dynamics. The proton-transfer phenomena were observed a few times during the simulation, although the proton is localized most of the time in the vicinity of the O8 and O5 proton-donor atoms. There is a clear correlation between the proton transfer (PT) events and the O1...O8 and O5...O4 interatomic distances. This could indicate that the PT reaction paths involve other deformation of the molecule, e.g., the coupling with the π electrons in the aromatic ring. The bridged protons are also able to stay for some time at the acceptor side (ca. 1 ps or 6 ps as it is shown in Figure 8), which is consistent with the model of double-well potential. The barrier is now frequently traversed due to increased thermal motions. The coupling between the bridges is visible: the proton jump occurs almost simultaneously in both bridges (panel a and b). We have studied the correlation between proton jumps in the hydrogen bridges, see Figure 9. The lines denote O(donor)-H distances in the two bridges. It is seen that the proton jumps are not ideally simultaneous. The first jump is followed by the jump in the second bridge, with some delay ranging from two to ca. 10 periods of the OH vibration. This delay, from 20 to 100 fs, is necessary for the propagation of the distortion through the aromatic system. Then, the situation of protons on the opposite sides, as discussed in the previous section (see Figure 2), leads to the point where the barrier to the complete double proton transfer is only ca. 6 kJ/mol, so the barrier is crossed and reaction is completed very fast.

The CPMD results showed the cooperation of the non-covalent intramolecular forces in Naphthazarin. It is visible that the bridged protons prefer to stay on the same side in the molecule. The crystalline phase simulation at 60 K provided a very similar picture of the bridged protons dynamics comparing to the gas phase results, see Figure S5 (panels c and d). The OH distances have not exhibit significant elongation. The protons are localized on the proton-donor side. Figure 8, panels c and d, presents time-evolution of bridged protons at 300 K. The external forces influence on the protons mobility was detected. The PT phenomena events were observed, but they are less frequent than in the gas phase. The protons are attached to the proton-acceptor atoms for shorter periods of time. There is a visible correlation in the hydrogen bridges dynamics and cooperativity of non-covalent internal forces. In Figure S6 we present the bridged protons dynamics from two stacked molecules from the unit cell as a result of the CPMD solid state simulations. There is no correlation in the protons dynamics in the two molecules. Therefore, we can conclude that our CPMD simulations showed the cooperativity between intramolecular non-covalent forces, but the solid state study revealed their competition.

**Figure 10:**
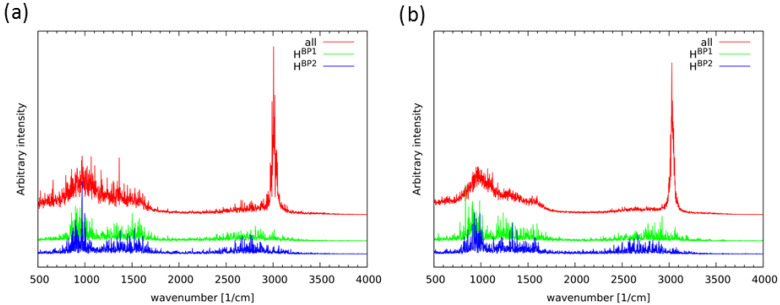
Atomic velocity power spectra resulting from the CPMD simulation at 300K, (**a**) in the gas phase, (**b**) in the solid state. Red line: the all-atom feature, related to the vibrational spectrum, green and blue lines—contributions of the bridge protons.

The dynamics of the bridged protons is directly connected with their spectroscopic features in the IR spectra. The vibrational analysis was performed in the gas phase as well as in the crystalline phase. In addition, the temperature influence on the spectroscopic signatures was investigated at 60 K and 300 K respectively. The analysis was performed on the basis of power spectra obtained as a result of Fourier transformation of autocorrelation function of atomic velocity. The power spectra enabled the spectra decomposition into atomic contributions, but with loss of the ability to reproduce infrared intensity; amplitudes of atomic motions are recovered instead. However, the application of the approach was dictated by the possible detection of the individual bridge proton contribution to the IR computed spectra. At this point we have to remind, that the CPMD approach treats atomic nuclei classically therefore we do not discuss issues related to the tunnelling or other phenomena. In the case of strongly anharmonic hydrogen bridges stretching, the shape of the spectral features was reproduced correctly, but with shifted positions of the maxima comparing to experimental data and other theoretical works [27,35]. The experimentally identified centers of υOH/OD and γOH/OD stretching modes were found in Naphthazarin at 3060/2200 cm${}^{-1}$ and 793/560 cm${}^{-1}$ respectively [27]. As it is shown below, our computational based CPMD data are in good agreement with experimental and computed IR spectra reported in Refs. [27,35]. The increased red-shifting is a result of combination of the used DFT plane-wave approach and CPMD-related “orbital drag” effect (aggravated by strong anharmonicity of the PES for this mode). The vibrational analysis performed at 60 K in the gas phase, showed two absorption regions for the bridged protons: 800–1700 cm${}^{-1}$ and 2500–2800 cm${}^{-1}$, centered at 2700 cm${}^{-1}$. It is important to stress that for both bridged protons the OH stretching regions were detected to be equal. The computed IR spectra are presented in Figure S7. The gas phase power spectrum obtained as a result of CPMD simulation at 300 K is shown in Figure 10. Two absorption regions were detected as well, but they are broader due to the thermal effects. The OH stretching regions were found to be at 650–1800 cm${}^{-1}$ and 2350–3250 cm${}^{-1}$. The solid state spectra computed at 60 K indicated two absorption regions as well, but they were not equal for the bridged protons. The OH stretching for the H${}^{BP1}$ was identified between 800–1700 cm${}^{-1}$ and 2700–3100 cm${}^{-1}$ with center at 2850 cm${}^{-1}$. The second bridged proton H${}^{BP2}$ covered absorption regions between 800–1700 cm${}^{-1}$ and 2400–2850 cm${}^{-1}$, centered at 2650 cm${}^{-1}$. The so called “cold dynamics” gave us more detailed and deeper insight into hydrogen bridges motions, see Figure S7 (panel b). There is visible oscillation symmetry break as a result of the external forces presence. We can see a shift in absorption values between the two bridged protons. Therefore the low temperature simulations provided an accurate description of the environmental effects influence on the spectroscopic signatures. The solid state spectrum obtained at 300 K is presented in Figure 10 (panel b). Similarly to above reported results, two absorption regions of the bridged proton were noticed. They were found at 600–1800 cm${}^{-1}$ and 2300–3300 cm${}^{-1}$ for the H${}^{BP1}$ and 600–1700 cm${}^{-1}$ and 2300–3200 cm${}^{-1}$ for the H${}^{BP2}$ respectively. Their centers are located at 2900 cm${}^{-1}$ and 2700 cm${}^{-1}$ indicating the oscillation symmetry break. This is in agreement with our CPMD solid state result at 60 K. The room temperature computed spectra have a broader absorption regions for the OH stretching due to the thermal motions. In addition, the comparison of the two-phases computation results revealed environmental effects influence on the spectroscopic signatures.

## 3. Computational Methodology

### 3.1. Static Density Functional Theory (DFT) and Diffusion Quantum Monte Carlo (DQMC)

The simulations were performed in a framework of Density Functional Theory (DFT) [70,71] and Møller-Plesset second-order (MP2) [72] perturbation theory. The neutronography data [18] of Naphthazarin and MP2 method served as references for the DFT results. The models of Naphthazarin, its isomers and transition state (TS) were prepared with assistance of the GaussView 5.0 [73] program. The geometry optimization of Naphthazarin molecule (see Figure 1 was performed using various functionals: CAM-B3LYP [74], APFD [75], M08-HX [76], HSE03 [77], TPSSh [78], N12-SX [79] and MP2 [72] method with application of 6-311+G(2d,2p) Pople’s-style basis set [80,81]. The harmonic frequencies were calculated to confirm that the obtained geometries of Naphthazarin correspond with the minima on the potential energy surface (PES) or transition state (TS). The proton reaction path in the O-H…O intramolecular hydrogen bonds was investigated by means of the scan with optimization method. The H…O (acceptor atom) distance was shortened with −0.05 Å increment (17 steps of the scan) while the remaining part of the molecule was optimized. Two-dimensional (2D) potential energy surface (PES) was generated. The simulations were performed at CAM-B3LYP/6-311+G(2d,2p) level of theory. The computations were carried out using the Gaussian 16, Rev. A.03 suite of programs [82]. Next, the PT reaction was designed and the energies of reactants, transition states (TS) and products were computed applying all listed above levels of theory. The thermodynamic data were obtained at the temperature of 298.15 K and pressure of 1 atmosphere as it is implemented in the Gaussian 16, Rev. A.03 package [82].

Following the energy barrier investigations for the asynchronous (single-particle) PT encompassing the Naphthazarin energy minima in C${}_{2v}$ and C${}_{2h}$ symmetry, also the D${}_{2h}$ structures related to the synchronous (double-particle) PT in the OH groups were calculated using various levels of theory including Diffusion Quantum Monte Carlo (DQMC) method. The structures were obtained as a result of the Car-Parrinello Molecular Dynamics (CPMD) geometry minimization [50] (see detailed description in the Section 3.3). The models were chosen in such a way that the double proton transfer could occur. Figure S2 shows models used for the energy minima and the proton-transfer barrier computation. The model on the left in Figure S2 is a minimum (with C${}_{2v}$ symmetry) while the model on the right shows transition state (TS) of higher symmetry. The fact that we investigated double (synchronous) transfer of two protons means that we need so far only two structures: the minimum and TS, because the minimum on the other site of the transfer is a symmetric equivalent of the first one.

The specific implementation of the Quantum Monte Carlo (QMC) technique used in this study is Fixed Node Diffusion Monte Carlo (FNDMC) [47,83]. The technique is accurate for both covalent and noncovalent interactions [84]. The FNDMC calculations are based on a standard Slater-Jastrow guide function with a Slater determinant built from Kohn-Sham B3LYP orbitals expanded into an all-electron triple-zeta STO basis set [85] and a variance-minimised Jastrow factor of an extended fourth-order Schmidt-Moskowitz type [86,87]. An improved drift-diffusion propagator was employed, and the time step was removed by time-step extrapolation [88].

Subsequently, the Harmonic Oscillator Model of Aromaticity (HOMA) [57] index was calculated as a descriptor of aromaticity changes in Naphthazarin during the scan of the proton reaction path H…O (acceptor atom) in the hydrogen bridge. The distorted geometries for the HOMA index were obtained at the CAM-B3LYP/6-311+G(2d,2p) level of theory. The aromaticity index was calculated based on the equation:

(1) $$\mathrm{HOMA}=1-\frac{\alpha }{n}\sum_{i=1}^{n}{\left(R_{i}-R_{opt}\right)}^{2}$$

where *n* is a number of bonds in aromatic ring taken into the summation, α is an empirical constant, *R*${}_{opt}$ is the optimal value of aromatic C-C bond and *R*${}_{i}$ is the calculated bond length.

The atomic nucleophilicity *f*${}^{-}$(*r*) and electrophilicity *f*${}^{+}$(*r*) indices were calculated at each point of the proton transfer reaction pathway between the Min_1 and Min_2 structures (OH bond distance elongation). The electron density population was computed using Hirshfeld method [89] implemented in the Gaussian 16, Rev. A.03 package [82] using CAM-B3LYP/6-311+G(2d,2p) level of theory for further application in Fukui functions computations [58]. The local atomic indices of nucleophilicity and electrophilicity were calculated using Fukui functions described as:

(2) $$f^{-}\left(r\right)=\rho_{N}\left(r\right)-\rho_{\left(N-1\right)}\left(r\right)$$

(3) $$f^{+}\left(r\right)=\rho_{\left(N+1\right)}\left(r\right)-\rho_{N}\left(r\right)$$

where $\rho_{N}$(*r*), $\rho_{\left(N-1\right)}$(*r*) and $\rho_{\left(N+1\right)}$(*r*) are electron densities, respectively for *N* electrons, *N* − 1 electrons and *N* + 1 electrons species. Continuing electronic structure and topology description of Naphthazarin, the Atoms In Molecules (AIM) theory [90] was employed. The wavefunctions for the analysis were obtained at CAM-B3LYP/6-311+G(2d,2p) level of theory using the Gaussian 09, Rev. C.01 suite of programs [91]. The atomic charges were computed for the proton transfer reaction model to reveal the changes in the electron density distribution upon the bridged proton position (Min_1, TS, Min_2). In addition, the topology of the molecule was studied by the electron density contour maps to detect Bond and Ring Critical Points (BCPs and RCPs). The electron density and its Laplacian changes upon the bridged proton position were examined as well. The AIMAll program [92] was applied for the analysis.

### 3.2. Symmetry-Adapted Perturbation Theory (SAPT)

The energy decomposition of the dimers of Naphthazarin was carried out using Symmetry-Adapted Perturbation Theory (SAPT) [49]. The set of investigated dimers is presented in Figure 7. The interactions energy was estimated for two sets of dimers: (i) extracted from the neutron diffraction experimental data measured at 60K [18], (ii) obtained as a result of quantum-chemical simulations at the CAM-B3LYP/6-311+G(2d,2p) level of theory [74,80,81]. The energy partitioning via SAPT method was performed at the SAPT2 level of theory [69] for both sets of studied dimers. The SAPT2 calculations for the DFT/CAM-B3LYP optimized structures were done with the aug-cc-pVDZ basis set [93]. The interaction energy computation at the SAPT2/aug-cc-pVDZ level of theory involved also the Basis Set Superposition Error (BSSE) estimation [94], dividing the investigated dimers into “monomers”. The SAPT calculations were performed using the Psi4 1.2.1 [95] program.

The SAPT method, which is considered a most standarised and expandable (see below for the description of the interaction term numbering scheme) approach to the analysis of intermolecular forces, divides an exact Hamiltonian of the system into the Hartree-Fock descriptions of monomers A and B, ${\hat{F}}_{A}$ and ${\hat{F}}_{B}$, correlation components interacting inside the monomers, ${\hat{W}}_{A}$ and ${\hat{W}}_{B}$, and the part describing interaction between monomers, $\hat{V}$. This yields the following formula:

(4) $$\hat{H}={\hat{F}}_{A}+{\hat{F}}_{B}+{\hat{W}}_{A}+{\hat{W}}_{B}+\hat{V}$$

This allows for the use of perturbational expansion with strict enforcement of partitioning between inter- and intra-monomer terms (hence the “Symmetry-Adapted” part of the method name). The resulting partial contributions are very characteristically labelled by a two-number system, e.g., $E_{exch}^{12}$ is a Pauli repulsion (exchange) term of the first order in the intermolecular operator and second order in the intramolecular correlation part. The SAPT terms can be amended by Hartree-Fock $\delta^{HF}$ or MP2 $\delta^{MP2}$ corrections which gather higher-order terms. The form used in this study includes the $\delta^{HF}$ term.

### 3.3. Car-Parrinello Molecular Dynamics (CPMD) in Vacuo and Crystalline Phases

The CPMD [50] computations were performed in the gas phase and in the solid state for 5,8-dihydroxy-naphthalene-1,4-dione using as a starting point experimental data reported for Naphthazarin C by Herbstein et al. [18]. The models used for simulations are presented in Figures S3 and S4 respectively. The energy minimization was carried out using the Hessian matrix of Schlegel [96] with the Perdew, Burke and Ernzerhof (PBE) functional [97] and the norm-conserving Troullier-Martins pseudopotentials [98]. The plane-wave kinetic energy cutoff was set to 80 Ry. The geometry minimization (in the crystalline phase) was performed with Γ-point approximation (i.e., using only Bloch eigenfunctions with zero reciprocal vector k to represent the periodic states in the crystal) [99]. The gas phase simulations were carried out in a cubic box with a = 14 Å, whereas the solid state model was constructed on the basis of experimental data (a = 7.664 Å, b = 7.304 Å, c = 15.16 Å, α = 90°, β = 114.60° and γ = 90°; the crystal structure Database Identifier is DHNAPH17 while the Deposition number is 1140006) [18]. The crystalline phase computations were performed with periodic boundary conditions (PBCs) and with real-space electrostatic summations for the eight nearest neighbors in each direction (TESR = 8). After the geometry optimization, Car-Parrinello molecular dynamics (CPMD) [50] simulations were carried out using the CPMD v3.11.1 suite of programs [100]. The time step was set to 3 a.u. and the fictitious electron mass parameter was equal to 400 a.u. for both phases. The CPMD computations were performed at 60 K and at 300 K and the Nosé-Hoover thermostat [101,102] was applied to control the assigned conditions. The initial parts of the obtained trajectories were taken as an equilibration and were not taken into account during the data analyses. The post-processing was performed for data obtained as results of gas and crystalline phases simulations at 60K and 300 K respectively. The CPMD trajectories were used for metric and infrared spectra (IR) analyses. The dynamics of the intramolecular hydrogen bonds was analyzed in detail and the temperature factor was taken into account to show its influence on the bridged protons movement. In addition, the correlations of the bridged proton dynamics were studied. The vibrational properties were analyzed in both temperatures as well. The Fourier transformation of the autocorrelation function of atomic velocity was applied for the computationally obtained infrared spectrum (IR) decomposition. The O-H stretching was plotted and analyzed separately to give deeper insight into spectroscopic signatures of the presence of the intramolecular hydrogen bonds. The hydrogen bridges dynamics was analyzed using scripts available in the VMD 1.9.3 suite of programs [103]. The Fourier transform power spectra of atomic velocity were computed using home-made scripts. The VMD [103], Gnuplot [104] and Mercury [105] programs were applied for the graphical presentation of computational models and obtained results.

## 4. Conclusions

The cooperativity/competition of diverse intra- and intermolecular forces was investigated in Naphthazarin C. We have studied the interactions in monomers, dimers and crystalline phase. The 2D PES confirmed that there are two energy minima. Therefore the proton transfer reaction path was designed and investigated to estimate the transition state (TS) and the second minimum energy. In order to reproduce the total energy, enthalpy and free Gibbs energy values, the DFT (with various functionals) and MP2 methods were applied. The results obtained based on CAM-B3LYP and MP2 methods are the most similar. Following the discussion of the energy barrier, we have additionally employed DQMC and CC methods with Dunning-style basis sets. The obtained energy barrier is above 50 kJ/mol. It is much higher comparing to DFT and MP2 results. The employment of DQMC and CC approaches provided an accurate PES description and up to our knowledge this is the first study where Quantum Monte Carlo was applied to Naphthazarin compound. The HOMA and Fukui functions results showed that the bridged proton position is able to influence significantly the aromaticity and reactivity of Naphthazarin. It is worth mentioning that the aromaticity in benzene ring decreases upon the proton migration to the acceptor side, but it is increasing in the 1,4-benzoquinone ring. The proton position in the hydrogen bridge is also responsible for the decrease/increase of the electrophilic and nucleophilic properties as it was shown by Fukui functions. In addition, an application of the AIM topological analysis conformed the presence of the two intramolecular hydrogen bonds being in agreement with earlier experimental and theoretical findings. Moreover, it provided information of the electron density qualitative changes upon the bridged proton position in the hydrogen bridge. The results of SAPT analysis performed for dimers extracted from the neutron diffraction and obtained as a result of DFT simulations showed that dispersion is a decisive force in the structure stabilization. The CPMD simulations performed at 60 K and 300 K revealed temperature-dependent features. The analysis of molecular dynamics of hydrogen bridges showed that there was not observed proton transfer phenomena at 60 K. However, the CPMD at 300 K, enabled us to notice proton transfer phenomena, moreover the proton were attached to the acceptor atom for ps period of time. The vibrational analysis performed in both temperatures for the whole molecule and bridged protons showed two regions with absorption with shifted centers showing the break of symmetry in the crystalline phase. The investigations of the bridged protons dynamics correlation showed that there is a cooperativity of forces in the molecule, but competition when we compare the dynamics of hydrogen bridges in two neighbouring molecules. 

## Figures and Tables

**Table 1:** Thermodynamic reaction path for the proton motion in the hydrogen bridge of Naphthazarin (asynchronous, single proton transfer path), for details see Figure 3. The energy characteristics was obtained based on DFT and MP2 method with 6-311+G(2d,2p) basis set.

Method	Thermodynamical Properties	Min_1	TS	Min_2
**CAM-B3LYP**	Δ*E* [kJ/mol]	0.0	29.9	25.3
**APFD**		0.0	22.1	18.9
**M08-HX**		0.0	40.1	31.2
**HSE03**		0.0	22.7	19.3
**TPSSh**		0.0	19.1	15.7
**N12-SX**		0.0	23.1	20.2
**MP2**		0.0	28.2	21.3
**CAM-B3LYP**	Δ*H* [kJ/mol]	0.0	28.7	25.0
**APFD**		0.0	20.8	18.5
**M08-HX**		0.0	38.7	31.0
**HSE03**		0.0	21.5	19.1
**TPSSh**		0.0	18.0	15.4
**N12-SX**		0.0	21.9	20.0
**MP2**		0.0	26.7	20.8
**CAM-B3LYP**	Δ*G* [kJ/mol]	0.0	31.2	25.7
**APFD**		0.0	23.3	19.4
**M08-HX**		0.0	41.5	31.7
**HSE03**		0.0	23.9	19.7
**TPSSh**		0.0	20.2	16.0
**N12-SX**		0.0	24.4	20.5
**MP2**		0.0	29.8	22.1

ΔE=ΣE(produts)−ΣE(reactants);ΔH=ΣH(produts)−ΣH(reactants);ΔG=ΣG(produts)−ΣG(reactants).

**Table 2:** Energy barrier for synchronous double proton transfer in Naphthazarin through the second-order saddle point (SSP) computed at various levels of theory including Diffusion Monte Carlo (DMC) method. The levels of theory are given using the usual convention level1//level2 indicating single point energy at level 1 for the structure optimized at level 2.

Both H on the Same Side	E(Min) [a.u.]	E(SSP) [a.u.]	Barrier [kJ/mol]
**B3LYP/TZPAE//MP2/aug-cc-pVTZ**	−685.84031	−685.82645	36.39
**DQMC//MP2/aug-cc-pVTZ**	−685.44024 ${}^{a}$	−685.41729 ${}^{b}$	60.26 ± 1.14 ${}^{c}$
**B3LYP/cc-pVTZ//B3LYP/cc-pVTZ**	−685.83187	−685.81670	39.82
**B3LYP/CBS//B3LYP/cc-pVTZ**	−685.91317	−685.89766	40.73
**B3LYP/aug-cc-pVTZ//B3LYP/cc-pVTZ**	−685.84182	−685.82685	39.31
**MP2/cc-pVTZ//MP2/cc-pVTZ**	−684.34634	−684.33145	39.08
**MP2/CBS//MP2/cc-pVTZ**	−684.69520	−684.68112	36.97
**MP2/aug-cc-pVTZ//MP2/cc-pVTZ**	−684.39847	−684.38402	37.95
**CCSD/cc-pVTZ//B3LYP/aug-cc-pVTZ**	−684.34813	−684.32221	68.04
**CCSD/cc-pVTZ//MP2/aug-cc-pVTZ**	−684.34723	−684.32092	69.07
**CCSD(T)/cc-pVTZ//B3LYP/aug-cc-pVTZ**	−684.48156	−684.46246	50.15
**CCSD(T)/cc-pVTZ//MP2/aug-cc-pVTZ**	−684.48149	−684.46208	50.97
**H atoms on opposite sides**	**E(Min) [a.u.]**	**E(SSP) [a.u.]**	**Barrier [kJ/mol]**
**B3LYP/cc-pVTZ//B3LYP/cc-pVTZ**	−685.82419	−685.81670	19.65

${}^{a}$ Statistical error: ±0.00030 a.u.; ${}^{b}$ Statistical error: ±0.00031 a.u.; ${}^{c}$ 1.14 kJ/mol is the statistical error of the DMC estimation of the barrier height.

**Table 3:** The SAPT energy partitioning at the SAPT2 level for the dimers of naphthazarin shown in Figure 7—extracted from the crystal X-ray data [18] (upper part) and reoptimized at the CAM-B3LYP/6-311+G(2d,2p) level of theory (lower part). All energy terms in kcal/mol. SAPT0 and SAPT2 are defined according to Ref. [69].

Dimer Type:	(a) Stacking	(b) Hydrogen Bond	(c) Hydrogen Bond	(d) Multipole
**Neutron diffraction structures**				
**Electrostatics**	−8.486	−4.195	−5.375	−2.440
**Exchange**	19.731	6.567	7.164	4.661
**Induction**	−3.071	−1.076	−1.285	−0.917
**Ind. $\delta E_{HF}^{\left(2\right)}$**	−2.010	−0.404	−0.436	−0.371
**Dispersion**	−24.054	−4.369	−4.426	−3.628
**SAPT0**	−16.270	−3.814	−4.769	−3.055
**SAPT2**	−15.880	−3.074	−3.922	−2.324
**DFT structures**				
**Electrostatics**	−2.752	−4.682	−4.780	−2.110
**Exchange**	4.724	5.806	6.015	2.385
**Induction**	−0.632	−1.064	−1.081	−0.522
**Ind. $\delta E_{HF}^{\left(2\right)}$**	−0.298	−0.373	−0.351	−0.169
**Dispersion**	−12.033	−4.043	−4.047	−2.587
**SAPT0**	−11.484	−4.877	−4.668	−3.571
**SAPT2**	−10.693	−3.983	−3.893	−2.833

## Data Availability

The data presented in the current study are available in the article and in the associated Supplementary Materials.

## Supplementary Materials

The following are available online at https://www.mdpi.com/article/10.3390/ijms22158033/s1.

## Abbreviations

The following abbreviations are used in this manuscript:

DFT	Density Functional Theory
DQMC	Diffusion Quantum Monte Carlo
CPMD	Car-Parrinello Molecular Dynamics
CC	Coupled Cluster
HOMA	Harmonic Oscillator Model of Aromaticity
AIM	Atoms In Molecules
SAPT	Symmetry-Adapted Perturbation Theory
NMR	Nucleic Magnetic Resonance
IR	Infrared spectrum
PES	Potential Energy Surface
TS	Transition State
SSP	Second-order Saddle Point
HB	hydrogen bond
PT	Proton Transfer
MP2	Møller-Plesset second-order perturbation theory
BCP	Bond Critical Point
RCP	Ring Critical Point
BSSE	Basis Set Superposition Error
2D	two-dimensional
