# Supplementary material for: Non-Covalent Forces in Naphthazarin—Cooperativity or Competition in the Light of Theoretical Approaches
Source: Int J Mol Sci. 2021 Jul 27;22(15):8033. doi: 10.3390/ijms22158033 (PMC8348774; doi:10.3390/ijms22158033)
Supplement: Supplementary file 1 [file ijms-22-08033-s001.zip › ijms-1318889-supplementary.pdf]

SUPPLEMENTARY INFORMATION

Non-covalent forces in Naphthazarin - cooperativity or competition in the light of theoretical approaches

Aneta Jezierska[1*], Kacper Błaziak[2,3*], Sebastian Klahm[4], Arne Lüchow[4], Jarosław J. Panek[1]

[1] University of Wrocław, Faculty of Chemistry, ul. F. Joliot-Curie 14, 50-383 Wrocław, Poland
[2] Faculty of Chemistry, University of Warsaw, ul. Pasteura 1, 01-224 Warsaw, Poland
[3] Biological and Chemical Research Center, University of Warsaw, Żwirki i Wigury 101, 01-224 Warsaw, Poland
[4] Institute of Physical Chemistry, RWTH Aachen University, 52056 Aachen, Germany

Table of content

I. **Figure S1.** Naphthazarin in its canonical (left) and tautomeric (right) forms with atoms numbering scheme applied in the study. The dotted lines indicate the presence of intramolecular hydrogen bonds.

II. **Figure S2.** Structures used for Diffusion Quantum Monte Carlo (DQMC) and static models simulations to calculate the energy minima and the energy barrier.

III. **Figure S3.** Schematic model of Naphthazarin cubic cell used for CPMD simulations in the gas phase.

IV. **Figure S4.** Schematic model of Naphthazarin molecules arrangement in the crystallographic unit cell used for CPMD simulations in the solid state, prepared on the basis of available neutron diffraction data [1].

V. **Figure S5.** Interatomic distances of atoms involved in the intramolecular hydrogen bond formation in Naphthazarin. The results of CPMD simulations at 60 K in the gas (**a**) and (**b**) and crystalline phases (**c**) and (**d**).

VI. **Figure S6.** The correlation between bridged protons in two Naphthazarin molecules. The results derived from the CPMD simulations in the solid state at 300 K for the stacked molecules in the unit cell [1]; mol. 1 indicates molecule 1 while mol. 2 – molecule 2.

VII. **Figure S7.** Fourier transforms of the atomic velocity autocorrelation function for Naphthazarin. The intensities are in arbitrary units, while the wave numbers correspond to the actual vibrational features of the system. The results were obtained from CPMD simulations in the gas (**a**) and crystalline (**b**) phases at 60 K.

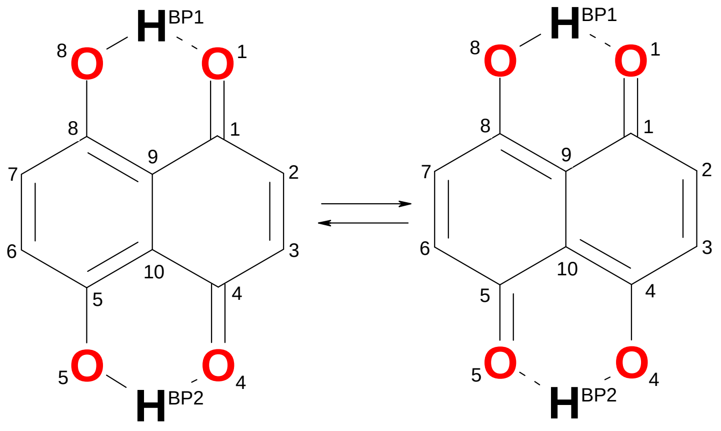

NA NA2

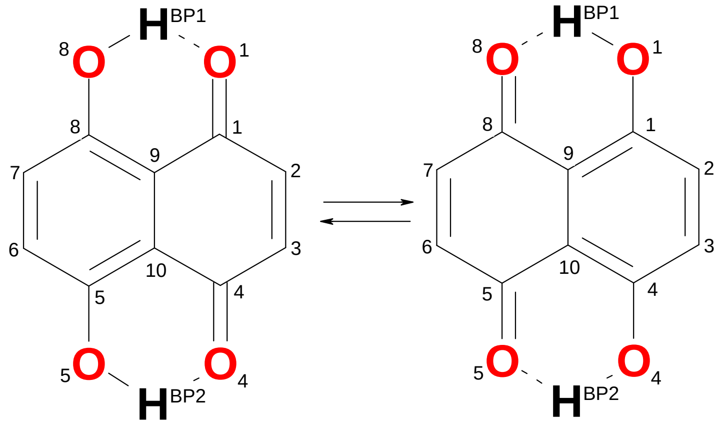

NA NA3

Figure S1. Naphthazarin in its canonical (left) and tautomeric (right) forms with atoms numbering scheme applied in the study. The dotted lines indicate the presence of intramolecular hydrogen bonds.

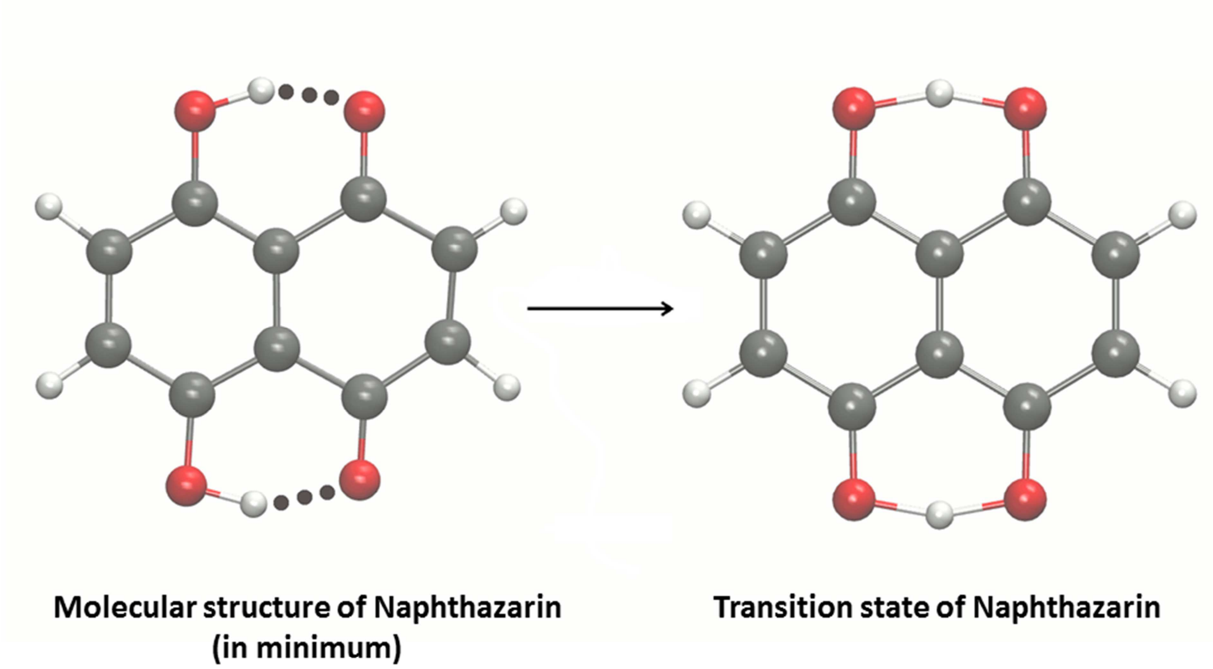

Figure S2. Structures used for Diffusion Quantum Monte Carlo (DQMC) and static models simulations to calculate the energy minima and the energy barrier.

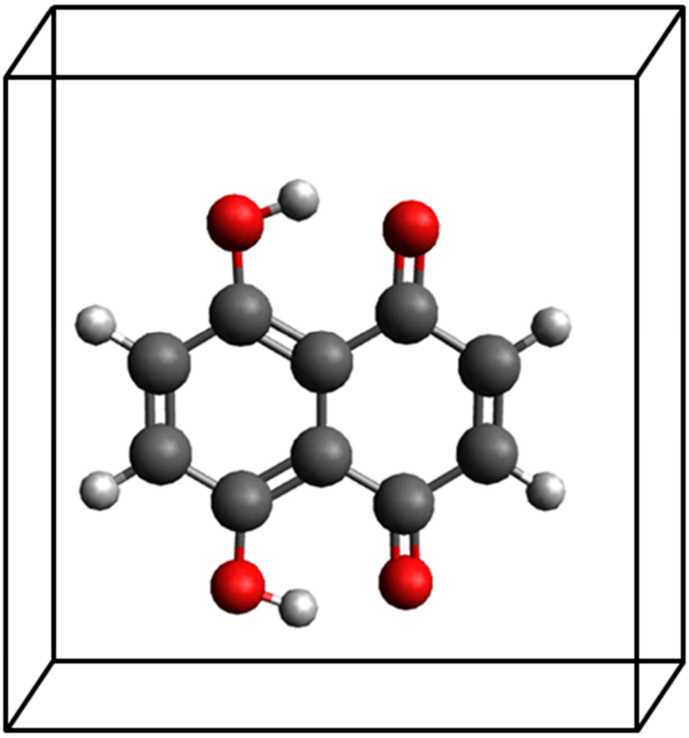

Figure S3. Schematic model of Naphthazarin cubic cell used for CPMD simulations in the gas phase.

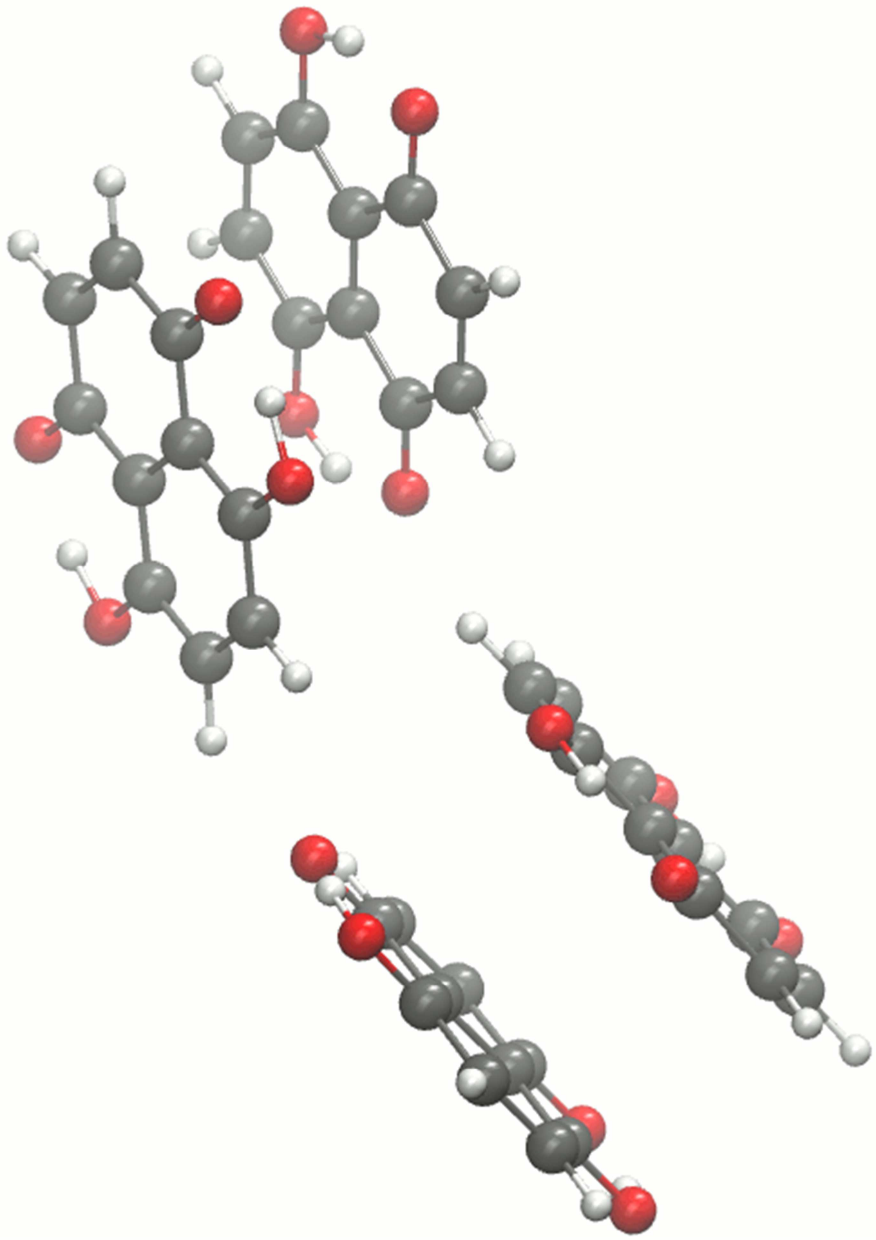

Figure S4. Schematic model of Naphthazarin molecules arrangement in the crystallographic unit cell used for CPMD simulations in the solid state, prepared on the basis of available neutron diffraction data [1].

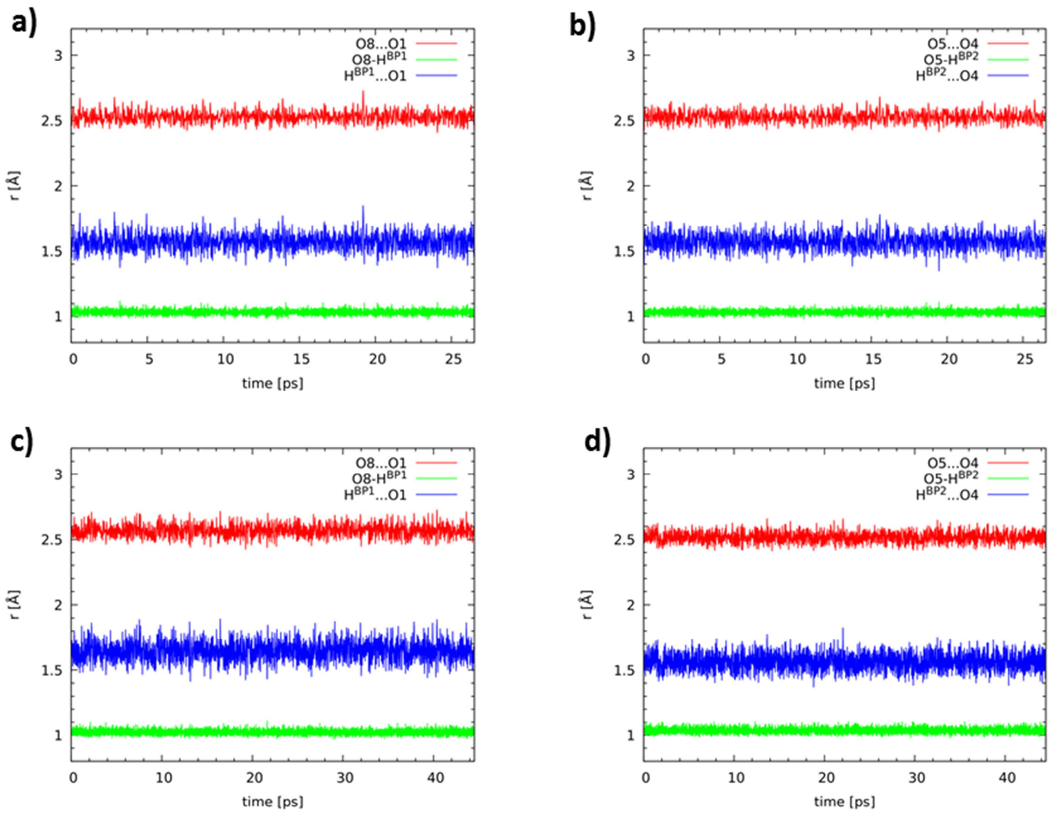

Figure S5. Interatomic distances of atoms involved in the intramolecular hydrogen bond formation in Naphthazarin. The results of CPMD simulations at 60 K in the gas (**a**) and (**b**) and crystalline phases (**c**) and (**d**).

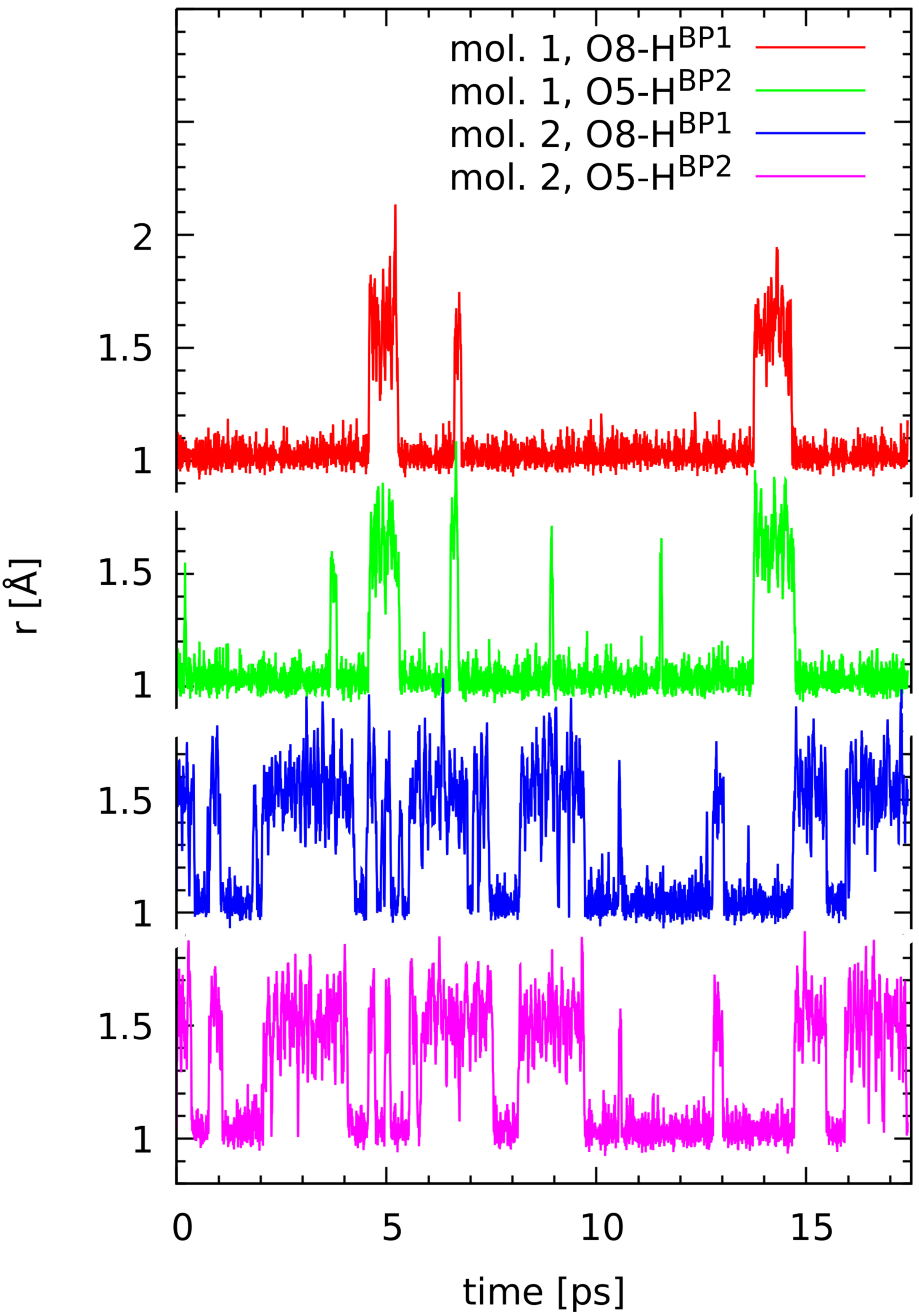

Figure S6. The correlation between bridged protons in two Naphthazarin molecules. The results derived from the CPMD simulations in the solid state at 300 K for the stacked molecules in the unit cell [1]; mol. 1 indicates molecule 1 while mol. 2 – molecule 2.

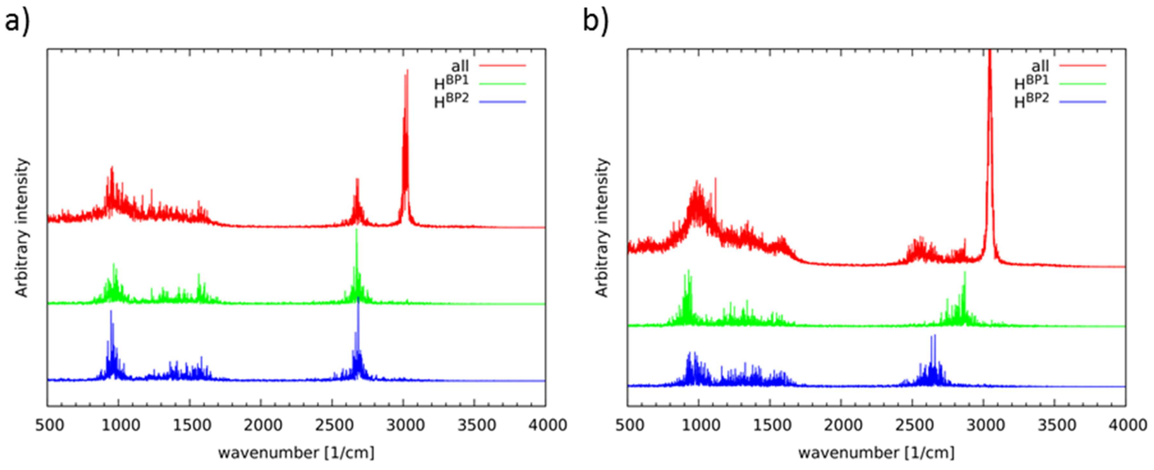

Figure S7. Fourier transforms of the atomic velocity autocorrelation function for Naphthazarin. The intensities are in arbitrary units, while the wave numbers correspond to the actual vibrational features of the system. The results were obtained from CPMD simulations in the gas (**a**) and crystalline (**b**) phases at 60 K.

References:

[1] F. H. Herbstein, M. Kapon, G. M. Reisner, M. S. Lehman, R. B. Kress, R. B. Wilson, W.-I. Shiau, E. N. Duesler, I. C. Paul, D. Y. Curtin. *Proc. R. Soc. London, Ser. A* **1985**, *399*, 295-319.
